# Magnolol Reduces Atopic Dermatitis-like Symptoms in BALB/c Mice

**DOI:** 10.3390/life14030339

**Published:** 2024-03-05

**Authors:** Ju-Hyun Lee, Dong-Soon Im

**Affiliations:** 1 Department of Biomedical and Pharmaceutical Sciences, Graduate School, Kyung Hee University, Seoul 02447, Republic of Korea; ljh0620@khu.ac.kr; 2 Department of Fundamental Pharmaceutical Sciences, Graduate School, Kyung Hee University, Seoul 02447, Republic of Korea

**Keywords:** *Magnolia officinalis*, atopy, magnolol, dermatitis, magnolia, anti-atopy, atopic dermatitis

## Abstract

In traditional Korean medicines, *Magnolia officinalis* is commonly included for the remedy of atopic dermatitis, and magnolol is a major constituent of *Magnolia officinalis*. Its pharmacological effects include anti-inflammatory, hepatoprotective, and antioxidant effects. Using BALB/c mice repeatedly exposed to 1-chloro-2,4-dinitrobenzene (DNCB), magnolol was evaluated in atopic dermatitis-like lesions. Administration of magnolol (10 mg/kg, intraperitoneal injection) markedly relieved the skin lesion severity including cracking, edema, erythema, and excoriation, and significantly inhibited the increase in IgE levels in the peripheral blood. A DNCB-induced increase in mast cell accumulation in atopic dermatitis skin lesions was reversed by magnolol administration, as well as a rise in expression levels of pro-inflammatory Th2/Th17/Th1 cytokines’ (IL-4, IL-13, IL-17A, IFN-γ, IL-12A, TARC, IL-8, and IL-6) mRNAs in the lymph nodes and skin (n = 5 per group). In lymph nodes, magnolol reversed DNCB’s increase in CD4^+^RORγt^+^ Th17 cell fraction and decrease in CD4^+^FoxP3^+^ regulatory T cell fraction. The results also showed that magnolol suppressed T cell differentiation into Th17 and Th2 cells, but not Th1 cells. Magnolol suppresses atopic dermatitis-like responses in the lymph nodes and skin, suggesting that it may be feasible to use it as a treatment for atopic dermatitis through its suppression of Th2/Th17 differentiation.

## 1. Introduction

Chronic inflammatory immune responses evoke atopic dermatitis, a skin disease [1]. Dysregulated immune responses are the leading cause, resulting from barrier defects, environmental factors, and genetic defects [1]. Atopic dermatitis reportedly affects 13.5~41.9% of the pediatric population, as per a global survey in 18 countries [2]. Widespread eczematous lesions are a cardinal characteristic of atopic dermatitis [3]. As in other atopic diseases, like allergic asthma, allergic rhinitis, and rhinoconjunctivitis, elevated IgE levels and type 2 immune responses are immunologic features of atopic dermatitis [4]. The Th2 response is not the only factor involved in the pathogenesis of atopic dermatitis, as the Th1 and Th17 responses are also deeply involved in the chronic phase of atopic dermatitis [5,6,7].

Administration of Gwakhyangjeonggi-san, a traditional Korean medicine, suppresses 1-chloro-2,4-dinitrobenzene (DNCB)-induced atopic dermatitis-like symptoms in mice [8]. Another Korean medicine, Pyeongwee-San (KMP6), also has a suppressive effect against atopic dermatitis in mice [9,10]. Gwakhyangjeonggi-san and KMP6 contain the cortex of *Magnolia officinalis*, in percentages of 8.7% and 21.5%, respectively. Magnolol (5, 5′-diallyl-2, 2′-dihydroxybiphenyl) is a main constituent in *Magnolia obovata* and *M. officinalis* [11]. The percentages of magnolol in *M. obovate* and *M. officialis* were found to be 4.4% and 2.6%, respectively [12]. The magnolol dosage was estimated to be 1.4 mg/kg since Gwakhyangjeonggi-san contained 8.7% of *M. officialis*, and 0.6 g/kg of it was administered in the atopic dermatitis study [8]. Similarly, because KMP-6 contains 21.5% of *M. officialis*, 1 g/kg was orally treated in the atopic dermatitis study [9], the dose of magnolol was estimated as 5.6 mg/kg. Therefore, three scientific references provided evidence of traditional use of two Korean medicines, Gwakhyangjeonggi-san and KMP-6, for atopic dermatitis therapy. Among the used plants, the bark of *Magnolia officinalis* is commonly included, and magnolol is the active constituent of *Magnolia officinalis.*

Previously, we found that honokiol (3,5′-diallyl-4,2′-dihydroxybiphenyl), a positional isomer of magnolol found in the bark of *Magnolia officinalis*, showed inhibitory activity on atopic dermatitis [10]. By administering honokiol, DNCB-induced mast cell accumulation and inflammation in skin tissues were significantly reduced [10]. Also, a significant reduction in inflammation-induced cytokines in skin and lymph nodes was observed after honokiol administration in combination with DNCB treatment [10]. In the present study, we investigated magnolol, a bioactive lignan found in the bark stripped from the roots, stems, and branches of the Houpu magnolia [13], a traditional Chinese medicine [14]. Magnolol has a variety of pharmacological effects, such as anti-anxiety, anti-cancer, anti-depressant, anti-inflammatory, antioxidant, and hepatoprotective properties [11,13,15,16,17,18,19,20]. Magnolol showed inhibitory effects on passive cutaneous anaphylactic reactions in mice [21] as well as on ovalbumin-induced allergic asthma by regulating T cell cytokines [22,23]. *Magnolia* has been commonly included in two traditional Korean herbal medicines (Gwakhyangjeonggi-san and KMP6) for atopic dermatitis treatment [9,10,21], and magnolol is the major constituent of *Magnolia*. However, it has not yet been explored in vivo whether magnolol is effective against atopic dermatitis. In the present study, thus, we evaluated whether magnolol could reduce atopic dermatitis symptoms using a murine DNCB-induced atopic dermatitis model and investigated its mechanism of action.

## 2. Results

### 2.1. Magnolol Suppresses Atopic Dermatitis-like Symptoms in the Ears of Mice

*Magnolia officinalis* has been included in two Korean herbal medicine concoctions for atopic dermatitis therapy [9,10,21], and magnolol is a major lignan found in the bark of the Houpu magnolia, a traditional Chinese medicine. Here, we evaluated the efficacy of magnolol against overall atopic dermatitis responses in a murine in vivo atopic dermatitis model caused by DNCB. The sensitization and challenges were made with DNCB, and magnolol or dexamethasone (DEX) was delivered 30 min before the DNCB treatment via intraperitoneal injection. We sacrificed the mice on day 49.

**Figure 1:**
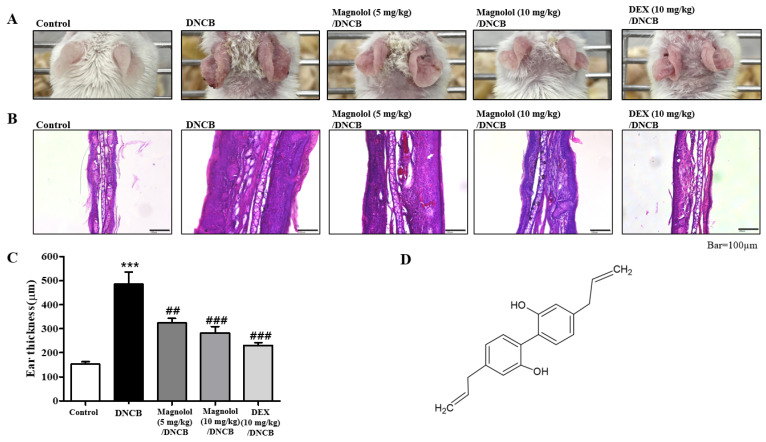
Effect of magnolol on 1-chloro-2, 4-dinitrobenzene [DNCB]-induced atopic dermatitis in ears of mice. (**A**) Photographs of the ears. (**B**) H&E staining. (**C**) Histogram of ear thickness. (**D**) Chemical structure of magnolol. *** *p* < 0.001 vs. the vehicle-treated group, ### *p* < 0.001, ## *p* < 0.01 vs. the DNCB-treated group. A magnification of ×200 was used.

As a result of DNCB, atopic dermatitis symptoms such as scaling, erythema, and erosion in the ears were experienced (Figure 1A). As a result of magnolol treatment, atopic dermatitis symptoms were ameliorated in the ears of DNCB-induced mice, as well as cutaneous hyperplasia, which is a key feature of atopic dermatitis-like lesions. For each treatment group, we measured the average thickness of the skin. The ears of magnolol-treated atopic dermatitis mice showed less swelling than their control counterparts (Figure 1A). A high level of epidermal hyperplasia is observed in atopic dermatitis skin lesions, along with an accumulation of immune cells. Hematoxylin and eosin (H&E) staining showed the anti-atopic dermatitis effects of magnolol (Figure 1B). Histologically, the infiltration of inflammatory cells into the dermis (exocytosis) and thickening of the epidermis (acanthosis) were reproduced in atopic dermatitis mice. In magnolol-treated atopic dermatitis mice, acanthosis and exocytosis were significantly suppressed (Figure 1B). Figure 1B shows a significantly lower ear thickness in magnolol-treated atopic dermatitis mice than in control atopic dermatitis mice. The measurement of ear thickness showed marked effects of magnolol, which were similar to the effects of DEX-treated mice (positive control) (Figure 1C). Figure 1D shows the chemical structure of magnolol.

**Figure 2:**
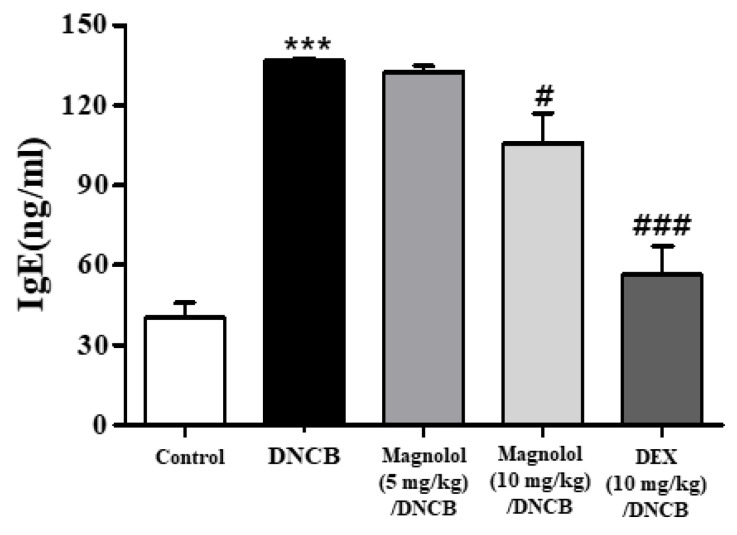
Effect of magnolol on the levels of serum immunoglobulin E. An ELISA was used to measure serum IgE levels. *** *p* < 0.001 vs. the vehicle-treated group, ### *p* < 0.001, # *p* < 0.05 vs. the DNCB-treated group.

The Th2 cytokines and IgE have a fundamental role in the pathogenesis of atopic dermatitis regardless of its high heterogeneity. A significant elevation in IgE levels is a cardinal character of atopic dermatitis and is linked with atopic dermatitis severity. Therefore, IgE levels in the serum obtained on day 49 were assessed by ELISA. Figure 2 shows that the basal level of serum IgE in the PBS group was about 40 ng/mL. A DNCB challenge, however, readily led to an increase in IgE to approximately 140 ng/mL. DNCB plus 10 mg/kg magnolol treatment, however, resulted in significant reductions in IgE levels compared to DNCB alone. It should be noted, however, that magnolol was less effective than DEX (Figure 2).

**Figure 3:**
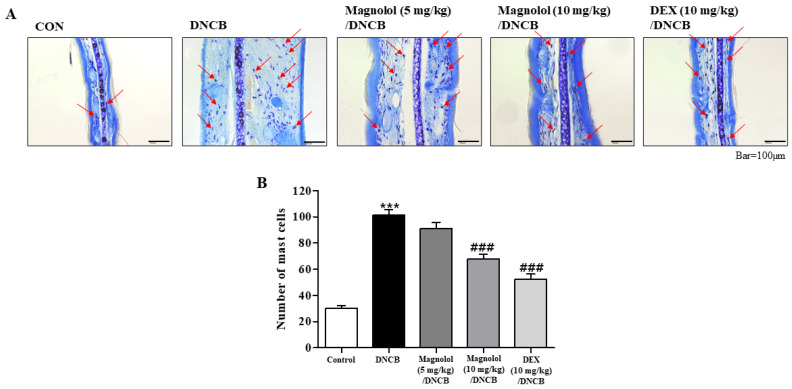
Magnolol reduces mast cell count in the ears. (**A**) Toluidine blue O staining of ear sections. Red arrows indicate mast cells. (**B**) Histogram of counts of mast cells (n = 5). *** *p* < 0.001 vs. the vehicle-treated group, ### *p* < 0.001 vs. the DNCB-treated group. ×200 magnification.

Mast cells are primarily involved in detrimental allergic disorders. Mast cell infiltration into the dermis was determined in toluidine blue O staining (Figure 3A). A significant increase in mast cell counts was observed following DNCB administration (Figure 3A). However, a dose-dependent decrease in the numbers was shown in atopic dermatitis mice treated with magnolol (Figure 3B). A similar decrease in mast cell numbers was shown in atopic dermatitis mice treated with DEX (Figure 3B).

**Figure 4:**
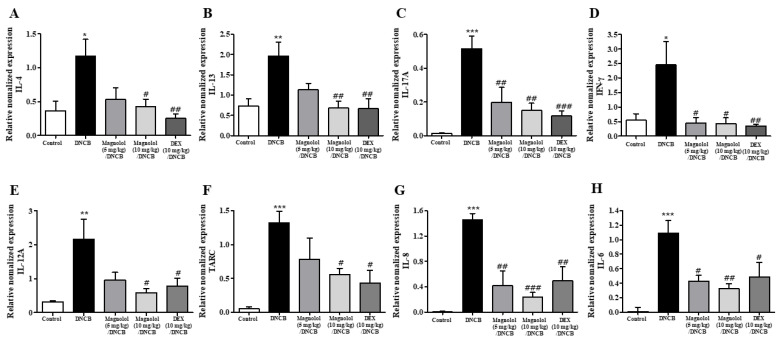
Effect of magnolol on the expression levels of pro-inflammatory cytokines mRNAs in the ears. (**A**) IL-4, (**B**) IL-13, (**C**) IL-17A, (**D**) IFN-γ, (**E**) IL-12A, (**F**) TARC, (**G**) IL-8, and (**H**) IL-6 mRNA expression levels in skin tissues (n = 5). *** *p* < 0.001, ** *p* < 0.01, * *p* < 0.05 vs. the vehicle-treated group, ### *p* < 0.001, ## *p* < 0.01, # *p* < 0.05 vs. the DNCB-treated group.

Atopic dermatitis is defined as an immune disorder driven by Th2 cells and characterized by elevated levels of interleukin (IL)-4, IL-13, and IL-5. In addition, the inflammatory pathogenesis of atopic dermatitis is influenced by Th1 and Th17 cytokines, interferons (IFN)-γ, IL-12A, and IL-17A. Thus, we measured pro-inflammatory cytokines from three different types of cells, Th2 (IL-4 and IL-13), Th1 (IFN-γ and IL-12A), and Th17 (IL-17A) [24]. An increase in mRNA expression levels of five cytokines was observed in the ear sections of atopic dermatitis-induced mice (Figure 4A–E). Pro-inflammatory cytokine gene expression was suppressed by magnolol or DEX (Figure 4A–E). As a chemokine, thymus and activation-regulated chemokine (TARC) plays an imperative role in the homing of skin-specific T cells [25], whereas IL-8 and IL-6 are involved in the recruitment of various cells into atopic dermatitis skin [26]. Therefore, the levels of TARC, IL-8, and IL-6 mRNA expression were measured. Each was significantly elevated in the ears of atopic dermatitis mice (Figure 4F–H). It was noted that both magnolol and DEX demonstrated a significant suppression of TARC, IL-8, and IL-6 mRNA expression levels (Figure 4F–H). Based on these results, magnolol appears to reduce the local inflammatory response caused by DNCB. All of these findings suggest that there is a significant attenuation of atopic dermatitis responses, hypertrophy, mast cell infiltration, and pro-inflammatory cytokine levels following magnolol administration in response to DNCB.

### 2.2. Magnolol Suppresses DNCB-Induced Enlargement of Lymph Node and Increases in mRNA Expression of Pro-Inflammatory Cytokines

**Figure 5:**
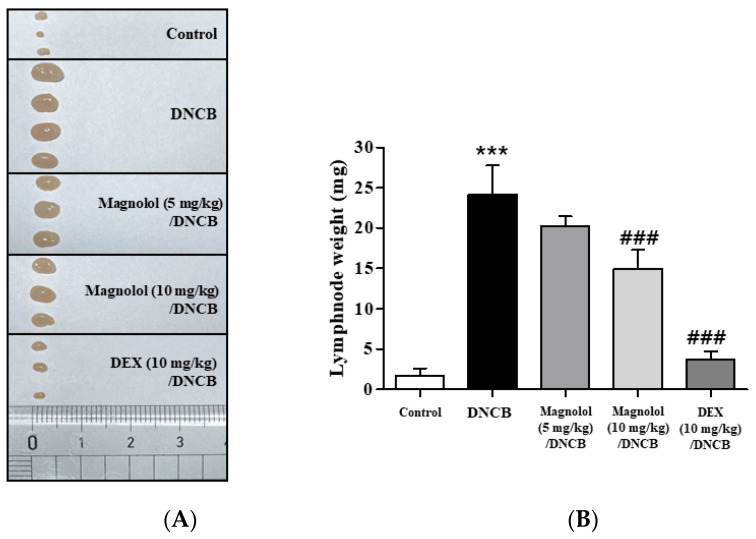
Effect of magnolol on immune responses induced by DNCB in lymph nodes. (**A**) Sizes of cervical lymph nodes. (**B**) Lymph node weight (n = 5). *** *p* < 0.001 vs. the vehicle-treated group, ### *p* < 0.001 vs. the DNCB-treated group.

Next, immune responses were investigated in the cervical lymph nodes. DNCB treatment enlarged the size of cervical lymph nodes (Figure 5), which was significantly suppressed dose-dependently following magnolol administration (Figure 5). A stronger suppression was observed in DEX-treated lymph nodes than in magnolol (Figure 5).

**Figure 6:**
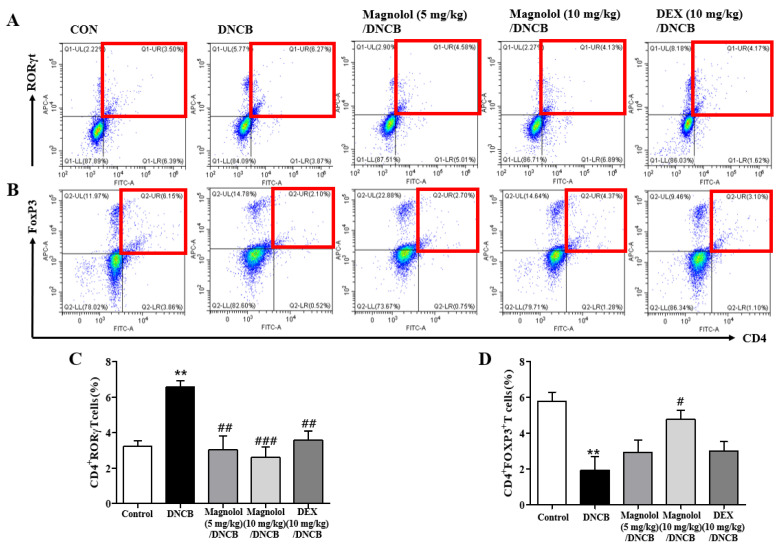
Suppressive effect of magnolol on the proportion of CD4^+^RORγt^+^ Th17 cells and CD4^+^FoxP3^+^ Treg cells in lymph nodes. (**A**) Dot plots of CD4^+^RORγt^+^ Th17 cells. (**B**) Dot plots of CD4^+^FoxP3^+^ Treg cells. (**C**) Percentage of CD4^+^RORγt^+^ Th17 cells. (**D**) Percentage of CD4^+^FoxP3^+^ Treg cells (n = 5). Double-positive cells are shown in the red-lined boxes. ** *p* < 0.01 vs. the vehicle-treated group, ### *p* < 0.001, ## *p* < 0.01, # *p* < 0.05 vs. the DNCB-treated group.

Flow cytometry determined the proportion of CD4^+^RORγt^+^ Th17 cells and CD4^+^FoxP3^+^ Treg cells in cervical lymph nodes, as these cells play key roles in the pathogenesis of atopic dermatitis, particularly in the chronic phase. In atopic dermatitis mice, CD4^+^RORγt^+^ Th17 cell numbers in lymph nodes increased, which was reversed by magnolol treatments (Figure 6A,C). As contrast, CD4^+^FoxP3^+^ Treg cells were decreased in atopic dermatitis mice, and magnolol restoration of this decrease was found (Figure 6B,D). A DNCB-induced increase in CD4^+^RORγt^+^ Th17 cell fraction was reversed by DEX administration in the atopic dermatitis model but not a decrease in CD4^+^FoxP3^+^ Treg cell fraction (Figure 6C,D).

**Figure 7:**
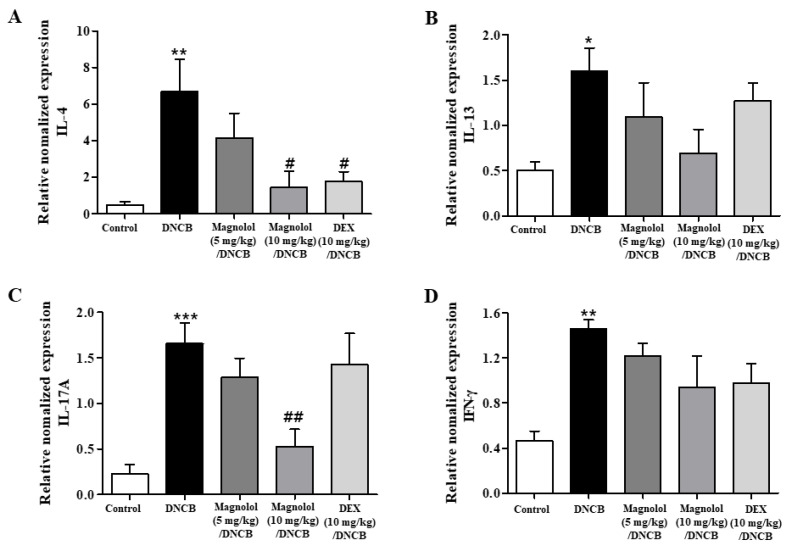
Effect of magnolol on the expression levels of pro-inflammatory cytokines mRNAs in lymph nodes. (**A**) IL-4, (**B**) IL-13, (**C**) IL-17A, and (**D**) IFN-γ mRNA expression levels in the lymph node tissues (n = 5). *** *p* < 0.001, ** *p* < 0.01, * *p* < 0.05 vs. the vehicle-treated group, ## *p* < 0.01, # *p* < 0.05 vs. the DNCB-treated group.

Additionally, we found that the levels of mRNA expression of Th2 (IL-4 and IL-13), Th1 (IFN-γ), and Th17 (IL-17A) cytokines were significantly increased in the cervical lymph nodes of atopic dermatitis mice induced with DNCB, as shown in Figure 7A–D. Figure 7A–D showed that magnolol treatment significantly suppressed the increased mRNA levels of these cytokines, especially IL-4 and IL-17A. Aside from reducing lymph node enlargement and suppressing inflammatory cytokines, magnolol reversed changes in CD4^+^RORγt^+^ Th17 and CD4^+^FoxP3^+^ Treg cell proportions caused by DNCB.

### 2.3. Magnolol Suppresses T Cells Differentiation into Th17 and Th2 Cells

**Figure 8:**
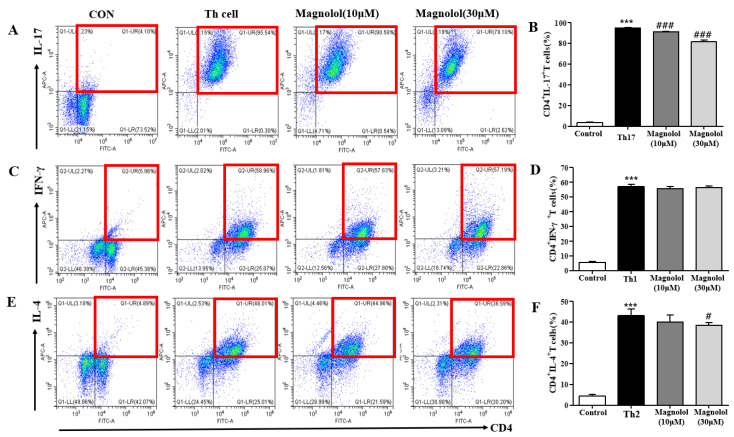
Suppressive effect of magnolol on T cell differentiation into Th17/Th1/Th2 cells. CD4^+^ T cells isolated from splenocytes were cultured in each differentiation media for Th17, Th1, or Th2 cells for 5 days in plates precoated with antibody to mouse CD3. (**A**,**C**,**E**) Flow cytometry results for (**A**) Th17 differentiation, (**C**) Th1 differentiation, and (**E**) Th2 differentiation. (**B**,**D**,**F**). Histograms of the cell percentage of (**B**) CD4^+^IL-17A^+^ cell population, (**D**) CD4^+^IFN-γ^+^ cell population, and (**F**) CD4^+^IL-4^+^ cell population (n = 5). Double-positive cells are shown as the red-lined boxes. *** *p* < 0.001 vs. the vehicle-treated group, ### *p* < 0.001, # *p* < 0.05 vs. the differentiation media-treated group.

A DNCB-induced increase in IL-17A expression was markedly suppressed by magnolol in the lymph nodes and skin, whereas a reduction in CD4^+^RORγt^+^ Th17 cell fraction in lymph nodes was reversed by magnolol. Therefore, we investigated whether magnolol could suppress the T cell differentiation into Th17 cells. As shown in Figure 8, magnolol treatment of Th17 differentiation media significantly suppressed the differentiation into IL-17A^+^ Th17 cells in a concentration-dependent manner in vitro (Figure 8A,B). Furthermore, we assessed the effects of magnolol on the differentiation into IFN-γ^+^ Th1 or IL-4^+^ Th2 cells and found that it did not affect IFN-γ^+^ Th1 cell generation (Figure 8C,D) but inhibited IL-4^+^ Th2 cell production (Figure 8E,F). In summary, magnolol treatment reduces the differentiation into IL-4^+^ Th2 cells and IL-17A^+^ Th17 cells.

## 3. Discussion

A murine atopic dermatitis model was evaluated to determine the effect of magnolol, since *Magnolia officinalis* is included in two traditional Korean herbal formulations for atopic dermatitis therapy (Gwakhyangjeonggi-san and KMP6) [9,10,11,21]. Previous studies revealed that magnolol contents in *M. obovate* and *M. officialis* were 4.4% and 2.6%, respectively [10]. As *M. officialis* constitutes 8.7% and 21.5% of Gwakhyangjeonggi-san and Pyeongwee-San (KMP-6), respectively, 53.7 mg/kg and 5.6 mg/kg of magnolol were estimated to be used in each atopic dermatitis experiment [9,10,21]. This study administered 5 mg/kg and 10 mg/kg magnolol in response to previous findings that 25–55 μM of magnolol inhibited lymphocytes and RBL-2H3 mast cells in vitro [21]. This study, for the first time, reports the anti-atopic efficacy of magnolol in a murine model. In the ears, mast cell accumulation and ear thickening were suppressed following magnolol treatment. In addition, magnolol treatment suppressed lymph node enlargement in magnolol-treated mice. Aside from suppressing serum IgE, magnolol decreased Th2/Th17/Th1 cytokines and chemokines in the ears and lymph nodes. An increase in CD4^+^FoxP3^+^ Treg cells and a reduction in CD4^+^RORγt^+^ Th17 cells in lymph nodes were observed in vivo following magnolol administration. Magnolol’s suppressive effects on T cell differentiation into IL-17A^+^ Th17 cells in vitro may be responsible for the decrease in CD4^+^RORγt^+^ Th17 cells in the lymph nodes in vivo.

Previous studies revealed that the contents of magnolol and honokiol in *M. obovate* were 4.4% and 1.17%, respectively, and in *M. officinalis*, 2.6% and 0.92%, respectively [10]. Considering that the contents of magnolol are higher than those of honokiol, magnolol may contribute mainly to the anti-atopic dermatitis efficacy of magnolia bark [27]. The potencies of magnolol and honokiol are quite similar in multiple parameters, including ear thickness, mast cell accumulation in skin tissues, serum IgE levels, size of lymph nodes, and inflammation-induced cytokines in the skin and lymph nodes [12]. The efficacy of magnolol on IL-17A expression levels is higher than that of honokiol with a statistically significant suppression by magnolol but not significant by honokiol [12]. Magnolol is an antioxidative molecule [28] and possesses antibacterial and anti-inflammatory activities [9,15,16,17,18,19,20], which could contribute to its anti-atopic dermatitis effects. Magnolol treatment suppressed significantly a lipopolysaccharide-induced increase in pro-inflammatory mRNA expression levels of TNF-α, MCP-1, NF-κB, and iNOS in the small intestine of rats [16]. Also, magnolol ameliorated an injury of mastitis tissues and reduced lipopolysaccharide-induced phosphorylation of p38, ERK, JNK, IκBα, and p65 in mouse mammary epithelial cells [17]. Magnolol significantly attenuated a lipopolysaccharide-induced lung injury via reducing immune cells in the bronchoalveolar lavage fluid and NF-κB signaling pathways in mice [18]. In dextran sulphate sodium-induced colitis mice, magnolol ameliorated disease activities index and suppressed expression levels of IL-1β, IL-12, and TNF-α via the regulation of NF-κB and peroxisome proliferator-activated receptor-γ pathways [19]. In imiquimod-induced psoriasis-like animal models, magnolol significantly inhibited pro-inflammatory cytokine expression [29], and it activated peroxisome proliferator-activated receptor-γ [30,31]. The anti-inflammatory properties of magnolol are attributed to several mechanisms, including redox-sensitive transcription factor NF-κB inhibition and PI3K/Akt signaling inhibition [29,32]. A reduction in eicosanoid mediator levels, including leukotrienes, as well as nitric oxide, TNF-α, and IL-4 appears to be required for magnolol to exert its effect [20,28,33]. Therefore, magnolol exerts its anti-inflammatory effects in multiple animal models, including acute lung injury, septic injury in ileum and mastitis, colitis, and psoriatic dermatitis, through suppressing NF-κB, peroxisome proliferator-activated receptor-γ pathways, and the suppression of pro-inflammatory mediators including leukotrienes, TNF-α, IL-4, IL-1β, IL-12, MCP-1, and iNOS.

Magnolol and magnolia bark extract inhibited the release of histamine from mast cells as well [27]. Mechanistically, the suppressive effects of magnolol on mast cells could be regarded as a possible mode of action underlying its anti-atopic dermatitis effects in vivo [27]. The anti-inflammatory actions of magnolol in murine models of asthma and allergic rhinitis have been demonstrated [22,23]. Furthermore, the suppressive effects of magnolol against IgE-mediated passive skin anaphylaxis [21] firmly support the findings of our study. As a mechanism of action of magnolol, we propose that magnolol possibly exerts its anti-inflammatory effects by suppressing Th17/Th2 differentiation and reversing changes in CD4^+^RORγt^+^ Th17 cell and CD4^+^FoxP3^+^ Treg cell populations in the lymph nodes. In the present study, we did not check whether magnolol affected T cell differentiation into FoxP3^+^ Treg cells. Given that magnolol reversed the DNCB-induced decrease in CD4^+^FoxP3^+^ Treg cell populations in the lymph nodes, magnolol may inhibit the suppressive effect of DNCB or enhance the naïve T cell differentiation into FoxP3^+^ Treg cells. Therefore, it is necessary to investigate whether magnolol affects T cell differentiation into FoxP3^+^ Treg cells. In addition, it would be worth investigating how magnolol differentially regulate T cell differentiation into each cell type. Considering the structural similarity between magnolol and honokiol, a comparative study on the effects of honokiol on T cell differentiation could help to elucidate the mode of action of magnolol.

Considering that magnolol was administered via intraperitoneal injection, it is worth investigating if the topical application of magnolol is as effective as intraperitoneal administration. A previous study on the psoriasis model already applied magnolol topically, and positive effects were observed [29], encouraging the topical application of magnolol. Furthermore, 2-*O*-acetyl-2′-*O*-methylmagnolol was found to be a potent and safe candidate for the treatment of cutaneous inflammation [34]. It was concluded that magnolol inhibited mast cell accumulation in a murine atopic dermatitis model and that the efficacy was likely due to the inhibition of pro-inflammatory cytokines in the skin (IL-17A, IL-4, IL-13, IFN-γ, and IL-12A) as well as chemokines (TARC, IL-8, and IL-6) in lymph nodes and epidermis. The anti-inflammatory properties of magnolol in the lymph nodes and epidermis may result from the inhibition of differentiation into Th17/Th2 cells. As a conclusion, this study suggests that magnolol is an active phytochemical with efficacy against atopic dermatitis and is a potential therapeutic constituent of *Magnolia officinalis* for atopic dermatitis therapy.

## 4. Materials and Methods

### 4.1. Chemicals

1-Chloro-2, 4-dinitrobenzene (DNCB), DEX (cat no. D1756, purity: ≥95% in HPLC), and magnolol (cat no. M3445, purity: ≥95% in HPLC) were purchased from Sigma-Aldrich (St. Louis, MO, USA).

### 4.2. BALB/c Mice

All experimental protocols for animal care and standard guidelines were performed in accordance with the rules and regulations of the Animal Ethics Committee of Kyung Hee University (KHSASP-22-407). In this study, Daehan Biolink provided 7-week-old male BALB/c mice housed in a room maintained at 22–24 °C and 60 + 5% relative humidity. The laboratory provided ad libitum chow and water.

### 4.3. Chemical-Induced Atopic Dermatitis Model and Magnolol Treatment

According to previous descriptions, DNCB causes atopic dermatitis-like symptoms [35]. The sensitization step was performed by spreading 1% DNCB on the dorsal skin on day 0, and the challenge step was performed by applying 0.3% DNCB to the ears every other day from day 7 through day 42. We randomly grouped mice into five groups (n = 5 per group): (1) PBS-treated controls, (2) DNCB-treated mice, (3) DNCB + magnolol (5 mg/kg)-treated mice, (4) DNCB + magnolol (10 mg/kg)-treated mice, and (5) DNCB + dexamethasone (10 mg/kg)-treated mice.

### 4.4. Serum Immunoglobulin E (IgE) Levels

Blood samples were collected from the mice on day 49. A centrifuge was used to obtain serum samples at 4 °C for 10 min. Samples were stored at 80 °C until use. Serum IgE levels were measured using sandwich ELISA with an IgE Mouse Uncoated ELISA Kit (cat. 88-50460-88; Invitrogen) and a 96-well plate (cat. 442404; Thermo Scientific, Waltham, MA, USA), according to the manufacturer’s instructions.

### 4.5. Mast Cell Count in the Skin

Detection of mast cells was facilitated by toluidine blue O staining. The counting of mast cells twice in 50 optical fields was conducted, and an average was calculated [35].

### 4.6. Histological Analysis of the Skin

On day 49, the ear skin of mice was evaluated for the severity of dermatitis. The ear tissues of mice were embedded in 10% formalin. After fixation, the ears were dehydrated in a 30% sucrose solution and embedded in O.C.T. compound. Using ImageJ software version 5, we measured skin thickness in the sections (8 μm) stained with H&E.

### 4.7. Quantitative Real-Time PCR

A total RNA sample was isolated from lymph nodes and skin using TRIzol (Invitrogen, Waltham, MA, USA). Promega’s MMLV reverse transcriptase was used to reverse transcribe the RNA into cDNA. For qRT-PCR, a CFX Connect Real-Time system (Bio-rad, Hercules, CA, USA) was used with Thunderbird Next SYBR qPCR Mix. There were 40 cycles of 95 °C for 30 s and 57 °C for 30 s in the PCR program. The last cycle at 95 °C for 30 s followed the cycle at 95 °C for 4 min. With the help of CFX Maestro Software version 2.3, the obtained data were analyzed using the 2^−∆∆Ct^ method. The primer sequences are listed in Table 1. Using GAPDH gene expression as the standard, results were normalized [36].

### 4.8. FACS Analysis

Cervical lymph nodes were separated, and single-cell suspensions were prepared using collagenase type II (Gibco, Grand Island, NY, USA). FITC-labeled rat antibodies against CD4 (cat. 11-0041-82, eBioscience) were used to stain single lymph node cells for 15 min at 4 °C to detect Th17 cell populations. It was then used to stain the permeabilized cells with anti-RORγt or anti-FoxP3 APC-labeled rat antibodies at 22 °C for one hour after fixation using IC fixation buffer. The cell sorting was performed on a CytoFLEX flow cytometer (Beckman Coulter, Brea, CA, USA).

### 4.9. Differentiation of Naïve T Cells into Th17/Th2/Th1 Cells

Naïve CD4^+^ T cells isolated from the spleens were differentiated into Th17, Th2, or Th1 CD4^+^ cells. Naïve CD4^+^ T cells in 12-well plates coated with anti-mouse CD3 were cultured in X-VIVO 15 (Lonza, Basel, Switzerland) for Th17 differentiation; in RPMI1640 media with recombinant mouse IL-12 (R&D 419-ML-010), recombinant human IL-2 (Peprotech 200-02), and anti-IL-4 (BioXcell BE0045) for Th1 differentiation; or in RPMI1640 media with recombinant human IL-2 (Peprotech 200-02), recombinant mouse IL-4 (Peprotech 214-14), and anti-mouse IFN-γ (BioXcell BE0055) for Th2 differentiation for 3 days [37]. The medium was replenished on day 3. The cells were collected on day 5 and analyzed for each differentiation using flow cytometry. Magnolol (10 and 30 µM) was added to each differentiation medium.

### 4.10. Statistics

A mean and standard error of mean (SEM) were calculated for all data (n = 5). An analysis of statistical significance was performed using version 5 of GraphPad Prism (GraphPad Prism). There was a *p* value of 0.05 set as the threshold for statistical significance. * *p* < 0.05, ** *p* < 0.01, and *** *p* < 0.001 vs. the vehicle-treated group, and # *p* < 0.05, ## *p* < 0.01, and ### *p* < 0.001 vs. the DNCB-treated group or differentiation media-treated group.

## Figures and Tables

**Table 1:** Quantitative real-time PCR primers.

Mouse Primers		Sequence
*Il-6*	Forward	5′-TTC TTG GGA CTG ATG CTG GT-3′
	Reverse	5′-CTG TGA AGT CTC CTC TCC GG-3′
*Il-8*	Forward	5′-AAC TCC TTG GTG ATG CTG GT-3′
	Reverse	5′-CCA GGT TCA GCA GGT AGA CA-3′
*Il-12A*	Forward	5′-GAA GCT CTG CAT CCT GCT TC-3′
	Reverse	5′-CAG ATA GCC CAT CAC CCT GT-3′
*IFN-g*	Forward	5′-CAC GGC ACA GTC ATT GAA AG-3′
	Reverse	5′-GTC ACC ATC CTT TTG CCA GT-3′
*TARC*	Forward	5′-AAT GTA GGC CGA GAG TGC TG-3′
	Reverse	5′-CAT CCC TGG AAC ACT CCA CT-3′
*Il-17A*	Forward	5′-AGC TGG ACC ACC ACA TGA AT-3′
	Reverse	5′-AGC ATC TTC TCG ACC CTG AA-3′
*Gapdh*	Forward	5′-AAC TTT GGC ATT GTG GAA GG-3′
	Reverse	5′-GGATGCAGGGATGATGTTCT-3′

## Data Availability

Data are contained within the article.

## References

[1] Labib A., Yosipovitch G. (2022). An evaluation of abrocitinib for moderate-to-severe atopic dermatitis. Expert Rev. Clin. Immunol..

[2] Silverberg J.I., Barbarot S., Gadkari A., Simpson E.L., Weidinger S., Mina-Osorio P., Rossi A.B., Brignoli L., Saba G., Guillemin I. (2021). Atopic dermatitis in the pediatric population: A cross-sectional, international epidemiologic study. Ann. Allergy Asthma Immunol..

[3] Paller A.S., Simpson E.L., Siegfried E.C., Cork M.J., Wollenberg A., Arkwright P.D., Soong W., Gonzalez M.E., Schneider L.C., Sidbury R. (2022). Dupilumab in children aged 6 months to younger than 6 years with uncontrolled atopic dermatitis: A randomised, double-blind, placebo-controlled, phase 3 trial. Lancet.

[4] Graff P. (2022). Potential Drivers of the Atopic March-Unraveling the Skin-Lung Crosstalk. Ph.D. Thesis.

[5] Kim J.Y., Jeong M.S., Park M.K., Lee M.K., Seo S.J. (2014). Time-dependent progression from the acute to chronic phases in atopic dermatitis induced by epicutaneous allergen stimulation in NC/Nga mice. Exp. Dermatol..

[6] Koga C., Kabashima K., Shiraishi N., Kobayashi M., Tokura Y. (2008). Possible pathogenic role of Th17 cells for atopic dermatitis. J. Investig. Derm..

[7] Muraro A., Lemanske R.F., Hellings P.W., Akdis C.A., Bieber T., Casale T.B., Jutel M., Ong P.Y., Poulsen L.K., Schmid-Grendelmeier P. (2016). Precision medicine in patients with allergic diseases: Airway diseases and atopic dermatitis-PRACTALL document of the European Academy of Allergy and Clinical Immunology and the American Academy of Allergy, Asthma & Immunology. J. Allergy Clin. Immunol..

[8] Son M.-J., Lee S.-M., Park S.-H., Kim Y.-E., Jung J.-Y. (2019). The effects of orally administrated Gwakhyangjeonggi-san on DNCB-induced atopic dermatitis like mice model. J. Korean Med. Ophthalmol. Otolaryngol. Dermatol..

[9] Jin S.-E., Moon P.-D., Koh J.-H., Lim H.-S., Kim H.-M., Jeong H.-J. (2011). Inhibition of chemokine, interleukin-8 expression in an atopic milieu by Pyeongwee-San extract (KMP6). Orient. Pharm. Exp. Med..

[10] Lee J.-H., Im D.-S. (2022). Honokiol suppresses 2, 6-dinitrochlorobenzene-induced atopic dermatitis in mice. J. Ethnopharmacol..

[11] Park J., Lee J., Jung E., Park Y., Kim K., Park B., Jung K., Park E., Kim J., Park D. (2004). In vitro antibacterial and anti-inflammatory effects of honokiol and magnolol against *Propionibacterium* sp.. Eur. J. Pharmacol..

[12] Fujita M., Itokawa H., Sashida Y. (1973). Studies on the components of Magnolia obovata Thunb. 3. Occurrence of magnolol and hõnokiol in M. obovata and other allied plants. Yakugaku Zasshi.

[13] Lee Y.J., Lee Y.M., Lee C.K., Jung J.K., Han S.B., Hong J.T. (2011). Therapeutic applications of compounds in the Magnolia family. Pharmacol. Ther..

[14] Zhang R., Zhang H., Shao S., Shen Y., Xiao F., Sun J., Piao S., Zhao D., Li G., Yan M. (2022). Compound traditional Chinese medicine dermatitis ointment ameliorates inflammatory responses and dysregulation of itch-related molecules in atopic dermatitis. Chin. Med..

[15] Zuo G.-Y., Zhang X.-J., Han J., Li Y.-Q., Wang G.-C. (2015). In vitro synergism of magnolol and honokiol in combination with antibacterial agents against clinical isolates of methicillin-resistant Staphylococcus aureus (MRSA). BMC Complement. Altern. Med..

[16] Yang T.-C., Zhang S.-W., Sun L.-N., Wang H., Ren A.-M. (2008). Magnolol attenuates sepsis-induced gastrointestinal dysmotility in rats by modulating inflammatory mediators. World J. Gastroenterol. WJG.

[17] Wei W., Dejie L., Xiaojing S., Tiancheng W., Yongguo C., Zhengtao Y., Naisheng Z. (2015). Magnolol inhibits the inflammatory response in mouse mammary epithelial cells and a mouse mastitis model. Inflammation.

[18] Yunhe F., Bo L., Xiaosheng F., Fengyang L., Dejie L., Zhicheng L., Depeng L., Yongguo C., Xichen Z., Naisheng Z. (2012). The effect of magnolol on the toll-like receptor 4/nuclear factor kappa B signaling pathway in lipopolysaccharide-induced acute lung injury in mice. Eur. J. Pharmacol..

[19] Shen P., Zhang Z., He Y., Gu C., Zhu K., Li S., Li Y., Lu X., Liu J., Zhang N. (2018). Magnolol treatment attenuates dextran sulphate sodium-induced murine experimental colitis by regulating inflammation and mucosal damage. Life Sci..

[20] Wang J.-P., Hsu M.-F., Raung S.-Z., Chen C.-C., Kuo J.-S., Teng C.-M. (1992). Anti-inflammatory and analgesic effects of magnolol. Naunyn-Schmiedeberg’s Arch. Pharmacol..

[21] Han S.J., Bae E.-A., Trinh H.T., Yang J.-H., Youn U.-J., Bae K.-H., Kim D.-H. (2007). Magnolol and honokiol: Inhibitors against mouse passive cutaneous anaphylaxis reaction and scratching behaviors. Biol. Pharm. Bull..

[22] Huang Q., Han L., Lv R., Ling L. (2019). Magnolol exerts anti-asthmatic effects by regulating Janus kinase-signal transduction and activation of transcription and Notch signaling pathways and modulating Th1/Th2/Th17 cytokines in ovalbumin-sensitized asthmatic mice. Korean J. Physiol. Pharmacol..

[23] Phan HT L., Nam Y.R., Kim H.J., Woo J.H., NamKung W., Nam J.H., Kim W.K. (2022). In-vitro and in-vivo anti-allergic effects of magnolol on allergic rhinitis via inhibition of ORAI1 and ANO1 channels. J. Ethnopharmacol..

[24] Lee J.E., Choi Y.W., Im D.S. (2022). Inhibitory effect of α-cubebenoate on atopic dermatitis-like symptoms by regulating Th2/Th1/Th17 balance in vivo. J. Ethnopharmacol..

[25] Hijnen D., de Bruin-Weller M., Oosting B., Lebre C., De Jong E., Bruijnzeel-Koomen C., Knol E. (2004). Serum thymus and activation-regulated chemokine (TARC) and cutaneous T cell–attracting chemokine (CTACK) levels in allergic diseases: TARC and CTACK are disease-specific markers for atopic dermatitis. J. Allergy Clin. Immunol..

[26] Kong L., Liu J., Wang J., Luo Q., Zhang H., Liu B., Xu F., Pang Q., Liu Y., Dong J. (2015). Icariin inhibits TNF-α/IFN-γ induced inflammatory response via inhibition of the substance P and p38-MAPK signaling pathway in human keratinocytes. Int. Immunopharmacol..

[27] Ikarashi Y., Yuzurihara M., Sakakibara I., Nakai Y., Hattori N., Maruyama Y. (2001). Effects of the extract of the bark of Magnolia obovata and its biphenolic constituents magnolol and honokiol on histamine release from peritoneal mast cells in rats. Planta Med..

[28] Shen J.-L., Man K.-M., Huang P.-H., Chen W.-C., Chen D.-C., Cheng Y.-W., Liu P.-L., Chou M.-C., Chen Y.-H. (2010). Honokiol and magnolol as multifunctional antioxidative molecules for dermatologic disorders. Molecules.

[29] Guo J.W., Cheng Y.P., Liu C.Y., Thong H.Y., Lo Y., Wu C.Y., Jee S.H. (2021). Magnolol may contribute to barrier function improvement on imiquimod-induced psoriasis-like dermatitis animal model via the downregulation of interleukin-23. Exp. Med..

[30] Dreier D., Latkolik S., Rycek L., Schnürch M., Dymáková A., Atanasov A.G., Ladurner A., Heiss E.H., Stuppner H., Schuster D. (2017). Linked magnolol dimer as a selective PPARγ agonist—Structure-based rational design, synthesis, and bioactivity evaluation. Sci. Rep..

[31] Dreier D., Resetar M., Temml V., Rycek L., Kratena N., Schnürch M., Schuster D., Dirsch V.M., Mihovilovic M.D. (2018). Magnolol dimer-derived fragments as PPARγ-selective probes. Org. Biomol. Chem..

[32] Tanaka K., Hasegawa J., Asamitsu K., Okamoto T. (2007). Magnolia ovovata extract and its active component magnolol prevent skin photoaging via inhibition of nuclear factor kappaB. Eur. J. Pharm..

[33] Hsu M.F., Lu M.C., Tsao L.T., Kuan Y.H., Chen C.C., Wang J.P. (2004). Mechanisms of the influence of magnolol on eicosanoid metabolism in neutrophils. Biochem. Pharm..

[34] Lin C.F., Hung C.F., Aljuffali I.A., Huang Y.L., Liao W.C., Fang J.Y. (2016). Methylation and Esterification of Magnolol for Ameliorating Cutaneous Targeting and Therapeutic Index by Topical Application. Pharm. Res..

[35] Kang J., Lee J.H., Im D.S. (2020). Topical Application of S1P(2) Antagonist JTE-013 Attenuates 2,4-Dinitrochlorobenzene-Induced Atopic Dermatitis in Mice. Biomol. Ther..

[36] Lee J.E., Im D.S. (2021). Suppressive Effect of Carnosol on Ovalbumin-Induced Allergic Asthma. Biomol. Ther..

[37] Flaherty S., Reynolds J.M. (2015). Mouse naive CD4+ T cell isolation and in vitro differentiation into T cell subsets. JoVE (J. Vis. Exp.).

